# Detection and treatment of cerebral toxoplasmosis in an aplastic pediatric post-allogeneic hematopoietic cell transplant patient: a case report

**DOI:** 10.1186/s12879-021-06650-2

**Published:** 2021-09-10

**Authors:** Danielle Brewer, Margaret L. MacMillan, Mark R. Schleiss, Satja Issaranggoon Na Ayuthaya, Jo-Anne Young, Christen L. Ebens

**Affiliations:** 1 Medical School, University of Minnesota, Minneapolis, MN USA; 2 Department of Pediatrics, Division of Blood and Marrow Transplantation and Cellular Therapy, University of Minnesota, Minneapolis, MN USA; 3 Department of Pediatrics, Division of Infectious Diseases, University of Minnesota, Minneapolis, MN USA; 4 Department of Medicine, Division of Infectious Diseases and International Medicine, University of Minnesota, Minneapolis, MN USA

**Keywords:** Toxoplasmosis, Allogeneic hematopoietic cell transplantation, Severe aplastic anemia, Immune mediated cytopenia, Cell-free DNA, Case report

## Abstract

**Background:**

Cerebral toxoplasmosis infection presents with non-specific neurologic symptoms in immunocompromised patients. With lack of measurable adaptive immune responses and reluctance to sample affected brain tissue, expedient diagnosis to guide directed treatment is often delayed.

**Case presentation:**

We describe the use of cerebrospinal fluid polymerase chain reaction and plasma cell-free DNA technologies to supplement neuroimaging in the diagnosis of cerebral toxoplasmosis in an immunocompromised pediatric patient following allogeneic hematopoietic cell transplantation for idiopathic severe aplastic anemia. Successful cerebral toxoplasmosis treatment included antibiotic therapy for 1 year following restoration of cellular immunity with an allogeneic stem cell boost.

**Conclusions:**

Plasma cell-free DNA technology provides a non-invasive method of rapid diagnosis, improving the likelihood of survival from often lethal opportunistic infection in a high risk, immunocompromised patient population.

## Background

Cerebral toxoplasmosis is a rare but serious complication of allogeneic hematopoietic cell transplantation (alloHCT). Caused by the protozoan parasite *Toxoplasma gondii*, toxoplasmosis most often results from reactivation of latent infection in immunocompromised patients [1]. It is one of the most common opportunistic infection of the central nervous system (CNS) [2], with greatest prevalence in those with acquired immunodeficiency syndrome (AIDS) [3]. The incidence of toxoplasmosis after alloHCT ranges from 0.3 to 9% [2, 4], with variation based on population seroprevalence. Although the incidence and treatment of toxoplasmosis in adult alloHCT patients has been reported extensively, few studies have focused specifically on cerebral toxoplasmosis in pediatric patients [5–17]. Furthermore, cerebral toxoplasmosis diagnosis is usually based on a combination of radiologic imaging abnormalities and clinical symptoms such as seizures, headaches, and altered mental status, non-specific findings contributing to delays in diagnosis and treatment [18]. This case reviews the successful management of cerebral toxoplasmosis in a pediatric alloHCT patient following diagnosis with the use of cerebrospinal fluid (CSF) polymerase chain reaction (PCR) and microbial cell free DNA (cfDNA) technology.

## Case presentation

A 13-year-old male with idiopathic severe aplastic anemia was treated with a human leukocyte antigen (HLA)-matched unrelated donor alloHCT on an Institutional Review Board-approved protocol with parental consent. His transplant course was complicated by Epstein-Barr virus (EBV) viremia (day + 21, successfully treated with rituximab), immune-mediated cytopenias versus inadequate graft function (beginning at day + 100, refractory to granulocyte-colony stimulating factor (GCSF), corticosteroids, intravenous immunoglobulin (IVIG), plasmapheresis and bortezomib), and right cervical lymphadenopathy concerning for EBV-post-transplant lymphoproliferative disease (day + 188, surgically excised, negative for infection or malignancy). With persistent pancytopenia, he required blood product transfusions and prophylactic anti-infective agents (valacyclovir, itraconazole, and intravenous pentamidine). Eight months after alloHCT, he was hospitalized locally for a severe gastrointestinal hemorrhage requiring superior mesenteric artery branch embolization.

**Fig. 1:**
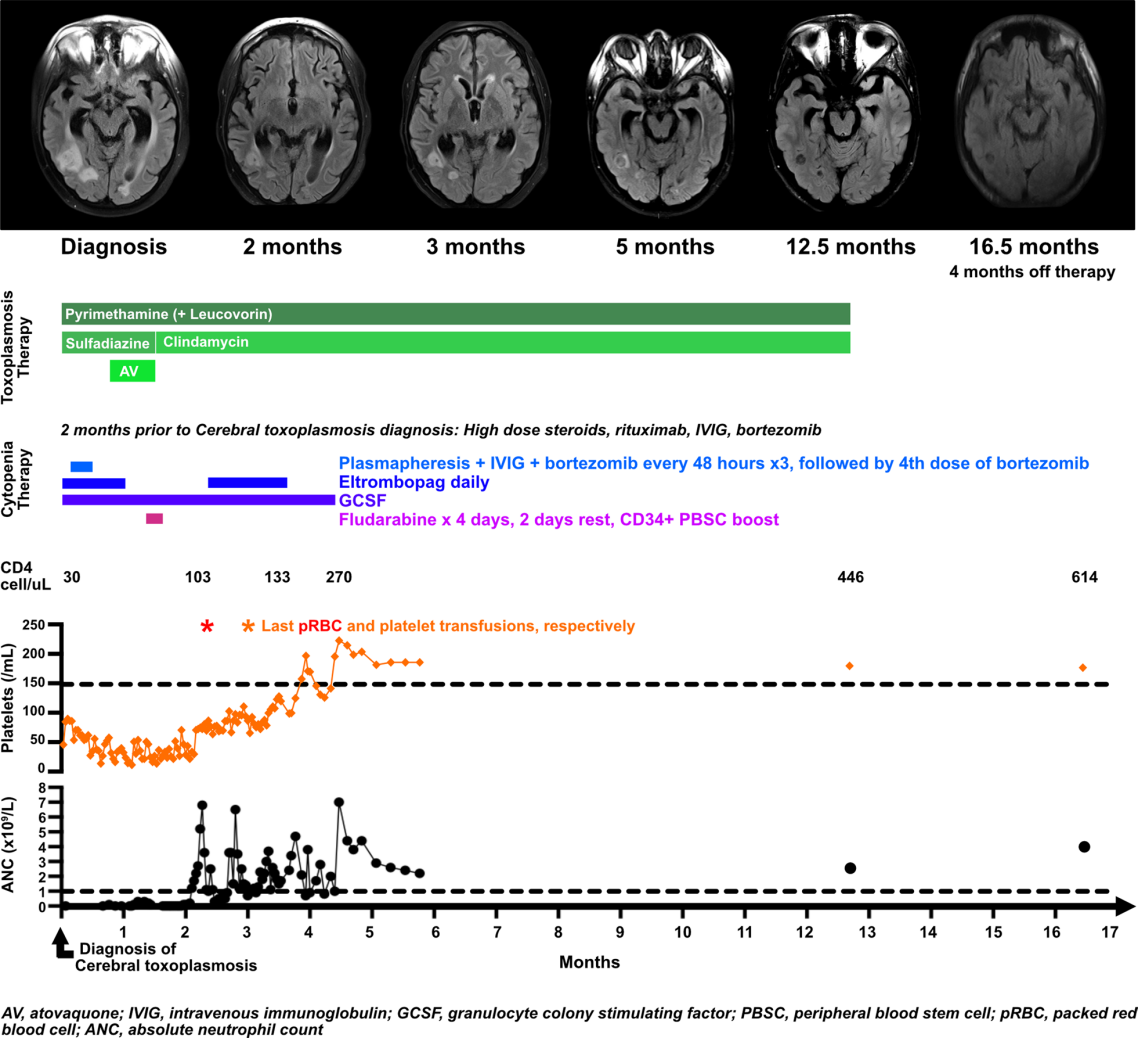
Resolution of cerebral toxoplasmosis with combined antibiotics and restoration of cellular immunity. Radiologic improvements in right posterior temporal and left occipital toxoplasmosis over time (axial fluid-attenuated inversion recovery 1.5 (16.5 months) or 3-T (all other) brain MRI images, are shown with a timeline of *Toxoplasma* directed antibiotics, interventions for presumed immune-mediated cytopenias, CD34 + PBSC boost, and blood laboratory findings (CD4 + lymphocyte count, platelet count, and absolute neutrophil count)

Nine months after alloHCT, he was readmitted to our hospital with refractory pancytopenia. He denied night sweats and weight loss, but endorsed 2 weeks of intermittent headaches. With no financial, cultural or social barriers to care, the patient was promptly evaluated. A bone marrow biopsy was hypocellular (5–10%), with 93% donor chimerism. On day 3 of hospitalization, his severe headache recurred, accompanied by somnolence, nausea, fever, and hypertension. Head computed tomography (CT) showed a curvilinear hyperdensity at the right parietal and occipital lobe junction. Brain magnetic resonance imaging (MRI), angiogram (MRA), and venogram (MRV) revealed numerous enhancing cerebellar and cerebral lesions with punctate microhemorrhages and surrounding vasogenic edema (Fig. 1). Compared to a previous brain MRI, third and 4th ventricle sizes were increased with accompanying ependymal enhancement concerning for possible hydrocephalus. Additionally, moderate stenosis of the distal transverse sinuses bilaterally raised concern for intracranial hypertension. Clinically, he had no focal neurological deficits and a normal ophthalmologic exam. Given his history, CNS EBV infection was initially suspected.

A lumbar puncture (LP) on hospital day 5 (alloHCT day + 304) revealed an elevated opening pressure of 38 cm H_2_O (normal 10–20) and CSF with 6 WBC/microliter (54% lymphocytes, 46% monocytes/macrophages), 8 RBC/microliter, glucose 39 mg/dL, and protein 112 mg/dL. *Toxoplasma gondii* was identified by CSF PCR and plasma cfDNA testing (5081 DNA molecules per microliter; Karius assay, Redwood City, CA), while serologies for *Toxoplasma* were negative. Given concurrent cytopenias and suspicion for alternative etiology, *Toxoplasma* therapy began only after these positive results (cfDNA testing returning in 2 days, CSF PCR in 4 days). Oral therapy with high dose pyrimethamine (200 mg loading dose followed by 75 mg once daily) with leucovorin rescue (50 mg daily) and sulfadiazine (1500 mg every 6 h daily) was initiated. While the patient was already on stress dosing hydrocortisone, three days of neuroprotective dexamethasone was provided within initiation of *Toxoplasma* therapy. Repeat *Toxoplasma* CSF PCR and plasma cfDNA testing was negative 2 weeks into treatment and remained so on future evaluations.

During the 3rd week of toxoplasmosis therapy, the patient required intensive care including 18 days of intubation/ventilation for an acute increase in somnolence and hypertension. While head CT and ophthalmologic exams were unchanged, his LP opening pressure was again elevated at 55 cm H_2_O. Improvement in mental status/alertness following the LP (closing pressure of 26.5) prompted initiation of acetazolamide and serial therapeutic LPs (16 times over 58 days). Atovaquone (1500 mg twice daily) was added when an MRI at 4 weeks of therapy (day + 337 post-alloHCT) showed decreased cerebral edema but unchanged toxoplasmosis lesions.

In the context of persistent cytopenias and poor graft function despite multi-modal therapy (Fig. 1), the patient received 4 days of immunosuppressive fludarabine followed by a CD34 + selected peripheral blood stem cell boost from his previous bone marrow donor (day + 349 after alloHCT). After 6 weeks of toxoplasmosis treatment showing both clinical and radiologic response, and to avoid bone marrow suppression after his stem cell boost, sulfadiazine was transitioned to oral clindamycin 600 mg 3 times/day for chronic maintenance therapy. One month after the stem cell boost, peripheral blood donor chimerism was 100% in the CD33 + myeloid compartment and 87% in the CD3 + lymphoid compartment. Transfusion independence was achieved at 42 days, eltrombopag discontinued at 60 days, and GCSF discontinued at 100 days. Fifty-five days following his stem cell boost—3 months of hospitalization—he was discharged on maintenance pyrimethamine and clindamycin. Adherence to oral therapies was monitored by nursing while inpatient and by the patient’s mother while outpatient. The patient himself reported no intolerance or adverse toxicities.

After 5 months of cerebral toxoplasmosis therapy, comprehensive neuropsychologic evaluations were completed. Compared to pre-alloHCT 14 months earlier, he displayed fine motor speed, dexterity and visuomotor integration deficiencies. From 6 to 12 months following cerebral toxoplasmosis diagnosis, his course was complicated by a single 30 s partial seizure. A brain MRI at 12.5 months of therapy revealed residual hypointense right posterior temporal lesions, resolution of associated vasogenic edema, and no new lesions. A bone marrow evaluation at that time was remarkable for 30–40% cellularity, trilineage hematopoiesis with no dysplasia and 98% donor contribution. With reassuring MRI findings and a CD4 count > 400 cells/microliter, toxoplasmosis therapy was discontinued. A 4 month off-therapy brain MRI was stable with no new lesions and interval improvement in mild ventriculomegaly.

## Discussion and conclusions

This case demonstrates the successful diagnosis and management of cerebral toxoplasmosis in a pediatric alloHCT patient. While seroprevalence of *Toxoplasma* exceeds 50% in some regions of the world, in both the United States and China (where this patient resided for 3 years), *Toxoplasma* is less common (~ 10%) [19, 20]. As such, surveillance for *Toxoplasma* is not routine prior to alloHCT at our institution and the serostatus of this patient was unknown. Risk factors for opportunistic reactivation included 4–6 months of preceding cytopenias and medication-associated immunosuppression from graft-versus-host disease prophylaxis, EBV treatment, and immune-mediated cytopenia therapies. Notably, routine prophylaxis against Pneumocystis jirovecii pneumonia with trimethoprim-sulfamethoxazole (TMP-SMX) until at least 1 year post-alloHCT and recovery of CD4 + lymphocyte count to > 200 cell/mm^3^ additionally protects against *Toxoplasma* reactivation and infection. However, to avoid further myelosuppression from TMP-SMX in this patient with concurrent cytopenias, his Pneumocystis jirovecii pneumonia prophylaxis had been transitioned to pentamidine, an agent with no activity against *Toxoplasma* [21]*.* Without standard alloHCT population recommendations, toxoplasmosis treatment and duration was based on U.S. Department of Health and Human Services “Guidelines for prevention and treatment of opportunistic infection in adults and adolescents with HIV” (available at https://aidsinfo.nih.gov2019).

PCR as a diagnostic tool for CSF samples of immunocompromised patients with suspected cerebral toxoplasmosis demonstrates wide variability in sensitivity [22–27]. Variations are attributable to laboratory variability, sample processing efficiency, and patient level differences in CSF protein and cellularity [27–29]. Regardless, CSF PCR remains less invasive than brain biopsy and provides rapid detection of parasite DNA. Moreover, CSF PCR expanded gene targets to detect *Toxoplasma* DNA [17, 28] are increasing accuracy of this methodology.

Microbial cfDNA sequencing technology provides a novel, non-invasive approach to the diagnosis of thousands of infectious organisms [30], including detection of opportunistic infection in immunocompromised hosts [31, 32]. However, cfDNA studies to date are limited by small sample sizes, lack of control groups, and cohort heterogeneity. Clinical indications for this novel approach remain to be clearly established. There is no published medical literature reporting the use of cfDNA to identify cerebral toxoplasmosis in an immunocompromised host. Prior to CSF PCR and plasma cfDNA sequencing results, the infectious differential diagnosis for our teenage alloHCT patient’s brain lesions included a broad group of neurotropic viruses, fungi and parasites. In our case, cfDNA sequencing provided rapid evidence of cerebral toxoplasmosis despite negative blood serologies and ophthalmologic examination. Thus, cfDNA sequencing emerges as a useful adjunct to diagnosis for toxoplasmosis, particularly when tissue diagnosis is not feasible [33].

Of note, while *Toxoplasma* serologies are often useful to assess for prior or current immune response to infection, they are unreliable before adequate immune reconstitution after alloHCT. This particularly patient was profoundly immune suppressed from treatment of immune mediated cytopenias after alloHCT and had recently undergone plasmapheresis, further reducing the likelihood of production of circulating antibodies. Interpretation of positive serologies, had they been found, would also be challenging as he had recently received IVIG.

While mortality of cerebral toxoplasmosis in post-alloHCT patients is reported from 38 to 67% [34], little is known about long term sequelae in adult or pediatric survivors [14]. While promptly initiated on antibiotics, our patient only displayed definitive clinical improvement after a CD34 + stem cell boost restored the cellular immunity essential for *Toxoplasma* clearance. Clinical and radiographic signs of recovery persisted at follow-up 4 months following completion of maintenance antibiotics. Future studies exploring the incidence and outcomes of cerebral toxoplasmosis in pediatric post-alloHCT patients are needed.

### Patient perspective

Fortunately during the time I was most ill as a patient I don’t really remember how I felt in the hospital and only have hazy memories. However, as I began to heal I do have memories of some nurses that especially helped me laugh during this time. I also remember enjoying integrative healing therapies in the form of music, aromatherapy, and massages. I am currently doing great, finishing my Freshman year of high school, playing in fantasy sports leagues, and also relieved to not be on clindamycin anymore.

## Data Availability

Data sharing is not applicable to this article as no datasets were generated or analyzed during the current study. All relevant data are herein included.

## Abbreviations

HCT: Allogeneic hematopoietic cell transplantation
CNS: Central nervous system
AIDS: Acquired immunodeficiency syndrome
CSF: Cerebrospinal fluid
PCR: Polymerase chain reaction
HLA: Human leukocyte antigen
EBV: Epstein-Barr virus
GCSF: Granulocyte colony stimulating factor
IVIG: Intravenous immunoglobulin
CT: Computed tomography
MRI: Magnetic resonance imaging
LP: Lumbar puncture
CMV: Cytomegalovirus
cfDNA: Cell free DNA
TMP-SMX: Trimethoprim-sulfamethoxazole
